# Automatic Explainable Segmentation of Abdominal Aortic Aneurysm From Computed Tomography Angiography

**DOI:** 10.1109/access.2025.3620721

**Published:** 2025-10-13

**Authors:** MERJULAH ROBY, ABU NOMAN MD SAKIB, ZIJIE ZHANG, SATISH C. MULUK, MARK K. ESKANDARI, ENDER A. FINOL

**Affiliations:** 1 Department of Mechanical, Aerospace, and Industrial Engineering, The University of Texas at San Antonio, San Antonio, TX 78249, USA; 2 Department of Computer Science, The University of Texas at San Antonio, San Antonio, TX 78249, USA; 3 Department of Thoracic and Cardiovascular Surgery, Allegheny Health Network, Allegheny General Hospital, Pittsburgh, PA 15212, USA; 4 Feinberg School of Medicine, Northwestern University, Chicago, IL 60611, USA

**Keywords:** Abdominal aortic aneurysm, deep learning, image segmentation, computed tomography imaging, explainable AI

## Abstract

This work presents an automated deep learning (DL) based framework for segmenting abdominal aortic aneurysm (AAA) in contrast-enhanced computed tomography angiography (CTA) images, which was developed to support AAA screening and analysis. The framework includes a dynamic router that assigns image regions to three specialized U-Net models, each trained to handle different aspects of the segmentation. It was trained and validated on 9,080 images and tested on 1,560 images representative of 22 unique patients. The model accurately segmented both the aortic lumen and the outer wall, achieving dice scores (DS) of 0.9648 and 0.9615, intersection over union (IoU) scores of 0.9324 and 0.9264, and Hausdorff distance (HD95) percentile values of 1.3490 mm and 1.3670 mm, respectively. The fully automated system processes each image frame in approximately 17 ± 1 milliseconds, making it suitable for real-time use. In certain complex cases where improved clinical accuracy is required, non-uniform rational B splines (NURBS) were used to manually refine the segmentation. In these cases, the NURBS correction time ranges from 3 to 20 seconds per frame. The framework’s training and validation demonstrate its potential as a reliable tool for AAA detection and clinical decision-making. Future work should focus on integrating multimodal imaging and optimization of NURBS to further improve its accuracy and efficiency.

## I. INTRODUCTION

### A. BACKGROUND

An Abdominal Aortic Aneurysm (AAA) manifests itself as a dilation of the abdominal aorta that expands beyond 50% its normal diameter. If left untreated, it will continue to grow until it ruptures. Endovascular aneurysm repair (EVAR), a minimally invasive procedure, as described by Moll et al. [1], is the preferred method for AAA repair in the United States. AAA screening programs have shown that population-based screening significantly reduces AAA-related mortality, as indicated by several trials reviewed in a meta-analysis. The Viborg study reported a prevalence of 4.0%, while the Chichester study found it to be 7.7%, emphasizing the variation in different populations [2]. Screening programs are essential tools that have been proven to be effective in the detection of AAA and can reduce specific and overall mortality, especially in the male population [3], [4]. The detection of AAA in women has long been a topic of debate despite being currently indicated in an at-risk population and suggesting that the maximum diameter of the aneurysm indexed to body size is a reliable predictor of rupture [5].

Screening programs for AAA detection can be resource intensive and are not universally implemented in healthcare systems. CTA scans, often used to diagnose other conditions, incidentally detect AAA in approximately 1.9% to 8.3% of the cases. Darling et al. [6] highlighted that these incidental findings, while different from the dedicated screening results, are crucial for effective monitoring and elective AAA repairs. Effective therapy of AAAs still depends on early discovery and monitoring, even with improvements in medical imaging and surgical techniques.

### B. RELATED WORK

In recent years, several pipelines have been proposed for AAA segmentation, including semi-automated and automated methods. Each method has varying degrees of complexity and requires different levels of user interaction. These advances have led to the application of artificial intelligence (AI) and DL techniques to automatically assess aortic morphology from CTA images. Such developments help clinical experts better characterize AAAs, predict their progression, and anticipate perioperative complications [7].

Progress in machine learning (ML) and DL algorithms have improved aortic image segmentation by providing accurate, reproducible results and facilitating feature extraction. DL techniques make it easier to model complex correlations in large clinical imaging datasets [8]. Spinella et al. [5] validated a fully automated DL-based pipeline for the screening of AAA in 73 thoracoabdominal CTAs. Using a 2.5D convolutional neural network (CNN) architecture, the pipeline achieved an accurate segmentation of the aortic structures, with a high classification accuracy of 97% for AAA cases and 96% for controls. Lu et al. [9] introduced DeepAAA, a DL model to detect AAAs from CT scans. Trained on 321 examinations, the model achieved a sensitivity of 91% and a specificity of 95%. Habijan et al. [10] proposed an automatic segmentation method using a modified 3D U-Net network; the study compares the segmentation performance of the original 3D U-Net with the modified version and evaluates it on 19 volumetric CT images, achieving a Dice Similarity Coefficient (DSC) of 91.03%. In addition, the work discusses the impact of up-sampling versus deconvolution layers on the performance of both networks for this specific clinical application. Chandrashekar et al. [11] developed a DL pipeline that uses an attention-based U-Net architecture for automated segmentation of aortic structures from contrast-enhanced and non-contrast CT images. Trained on 75 CT scans, the model accurately identified thelumen and wall boundaries, achieving DSC scores of 93.2% for contrast-enhanced and 88.7% for non-contrast images). It showed strong correlations with manual measurements for key clinical metrics, such as maximum diameter and volume. Lyu et al. [12] tested two DL models, ARU-Net [13] and CACU-Net [14], to automatically segment AAAs from CT scans. Both models performed comparable to human experts, with CACU-Net being slightly more accurate. Using these models significantly reduced the time required to generate volume domains for computational hemodynamic analysis from approximately 2 hours to just 10 minutes. Kim et al. [15] proposed a DL model that predicts AAA growth using features such as vessel radius, thrombus thickness, blood flow stress, and past growth rates. It introduced a local patch-based CNN that transforms 3D features into 2D inputs, enabling learning from limited patient data. The model significantly outperformed existing methods, achieving high accuracy in predicting AAA shape and growth over a year. The aforementioned related work provides a strong rationale for the feasibility of automating AAA analysis in clinical workflows, although further validation is needed on larger datasets.

Fantazzini et al. [16] proposed a DL pipeline that uses multiview 2D CNNs to automatically segment the aortic lumen from CT angiography scans. It combined axial, sagittal, and coronal views to produce accurate 3D segmentations with DSC scores of nearly 0.93, outperforming single view methods. The method requires approximately 25 seconds per image and is effective for complex aortic structures. Their data set is small and single-centered, which may affect the model’s generalizability and robustness across diverse patient anatomies. Roby et al. [17] proposed an automated segmentation method for AAA using a patch-based dilated U-Net model applied to CTA images. The model leveraged dilated convolutions and patch-based processing to efficiently and accurately delineate the lumen and outer wall boundaries, outperforming traditional segmentation methods in both speed (17 ± 0.02 ms per image) and accuracy. It also incorporated Non-Uniform Rational B-Splines (NURBS) for refining the segmented boundaries, which proved beneficial in complex cases involving image artifacts. Extensive validation demonstrated the potential clinical utility of the method, closely aligning automated results with expert segmentations. Although the foregoing techniques and algorithms show promising performance, they are challenged to provide refined and accurate segmentations when faced with more complex input data, which may include thin walls, neighboring pixels with similar intensity values, and other complex features.

### C. CONTRIBUTION OF THE STUDY

To address the limitations of existing segmentation approaches and to improve robustness and accuracy in AAA boundary detection, we propose a novel explainable and automated segmentation framework. Our approach has three independently trained, specialized U-Net models with varying configurations, each expert at detecting different structural attributes of an AAA. These models operate in parallel, generating predictions that are analyzed using an error-learning router. The router integrates input images and expert predictions to classify and learn from segmentation errors. A robust weight router then learns spatially varying attention weights to adaptively fuse expert predictions. This Mixture-of-Experts (MoE) strategy enables accurate identification of both the lumen and the outer wall boundaries while maintaining explainability for clinical interpretation. The contributions of the present work are summarized as follows. We

- propose an explainable segmentation pipeline using three experts’ models, each trained on distinct configurations to identify different anatomical patterns of an AAA;
- introduce an error-learning router that identifies and classifies segmentation errors by analyzing expert predictions with the original input image;
- develop a robust weight router that learns per-pixel attention weights to combine expert predictions in a spatially adaptive and explainable manner;
- provide an accurate and explainable segmentation of both the lumen and the wall of AAA, which is important to assess the risk of rupture;
- conduct an exploratory analysis of different expert results along with the performance of the explainable router to enhance the segmentation robustness and model generalizability;
- and introduce techniques for precise quantification of lumen and outer wall boundaries, enhancing a detailed assessment of AAA severity for improved segmentation outcomes.

The remainder of this paper is organized as follows. Section II describes the proposed automated DL framework, including the dynamic router and specialized U-Net models. Section III presents the results, including segmentation performance, quantitative analysis, and explainability analysis. Section IV discusses the implications, limitations, and potential clinical applications of the framework. Finally, Section V provides a conclusion and outlines directions for future research.

## II. METHODOLOGY

**FIGURE 1.**
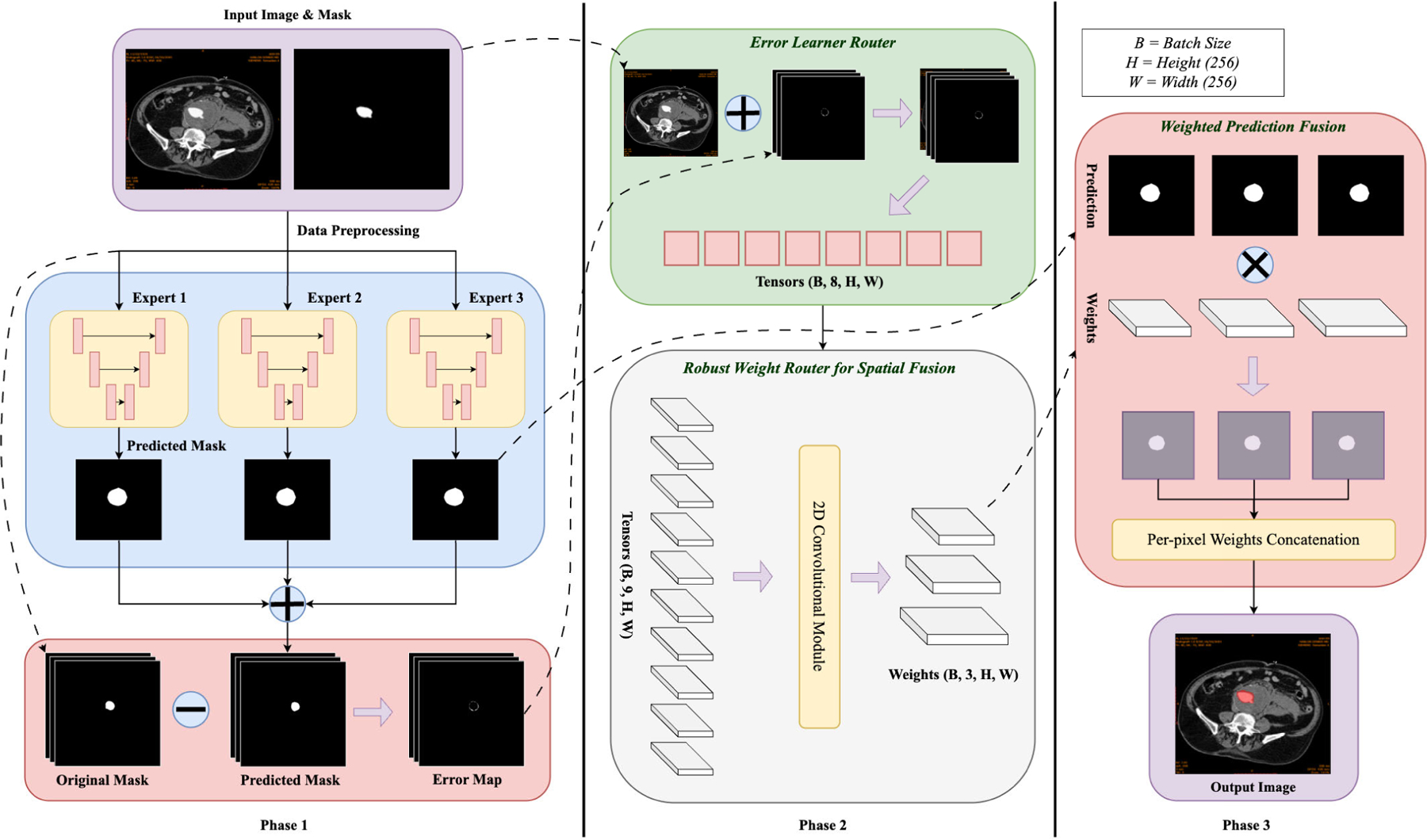
Block diagram of the proposed overall framework using a Mixture of Experts (MoE). Phase 1 encompasses data preprocessing, expert network predictions, and error map generation. Phase 2 illustrates the explainable routers: the Error Learner Router (top) and the Robust Weight Router (bottom). Phase 3 performs weighted prediction fusion to yield the final segmentation output.

The combination of three U-Net models and the router, with its loss functions, enables the explainable segmentation framework to address a wide range of challenges in clinical image segmentation. By dynamically routing input regions and focusing on complex areas using attention maps, the framework ensures robust segmentation performance in tasks such as tumor delineation, organ localization, and multimodal image fusion [19], [20], [21], [22]. Figure 1 shows a DL-based segmentation pipeline to process input images and generate segmentation masks. The pipeline begins with an input CTA image, processed by a router, which dynamically assigns different regions of the image to three specialized U-Net models, each focusing on a distinct aspect of the segmentation. The outputs of these models are then combined using attention weights generated by the router, which determine the contribution of each expert model. The router generates attention weights that determine the contribution of each expert model to the final prediction, allowing an adaptive combination of their outputs. Model training and implementation were performed using the UTSA ARC (Advanced Research Computing) cluster. The GPU nodes included two Intel CPUs with 24 cores each, four NVIDIA H100 GPUs with 80 GB memory per GPU, and 1 TB of RAM. This setup provided the computational power needed to handle high-resolution medical images and support the multi-model segmentation framework efficiently.

### A. DATA SET

The data set used in this study consists of 2D high-resolution CTA images collected retrospectively from two clinical centers: Allegheny General Hospital (Pittsburgh, PA) and Northwestern Memorial Hospital (Chicago, IL). Ethical clearance and Institutional Review Board (IRB) approvals were obtained prior to data collection. The axial CTA slices have a resolution of 512 × 512 pixels, with a slice thickness ranging from 1 mm to 3 mm. Ground truth annotations were generated using a semi-automated tool, AAAVasc (v. 0.168, The University of Texas at San Antonio), which delineates the lumen and vessel wall boundaries [18]. The data set includes 9,037 images for training and 1,560 images for testing. The latter comprises 22 unique AAA CTA exams.

### B. PROPOSED FRAMEWORK

To achieve accurate and explainable AAA CTA segmentation, we designed a modular framework that integrates multiple specialized models and adaptive routing mechanisms. The architecture is built to identify challenging regions in CTA images, leverage the strengths of individually trained models, and fuse their outputs in a spatially intelligent manner. The proposed segmentation framework consists of the following main components:

1. Data Preprocessing
2. Specialized Expert U-Net Models
3. Error Map Generation
4. Error Learner Router
5. Robust Weight Router for Spatial Fusion
6. Weighted Prediction Fusion

The complete architecture is shown in Figure 1 and each component is discussed in detail below.

#### 1) DATA PREPROCESSING

Each CTA image is first converted to grayscale format and resized to a uniform dimension of [1, H, W] to ensure compatibility across the network. The preprocessed images are the input to the three expert models and the subsequent routing modules.

#### 2) SPECIALIZED EXPERT U-NET MODELS

**FIGURE 2.**
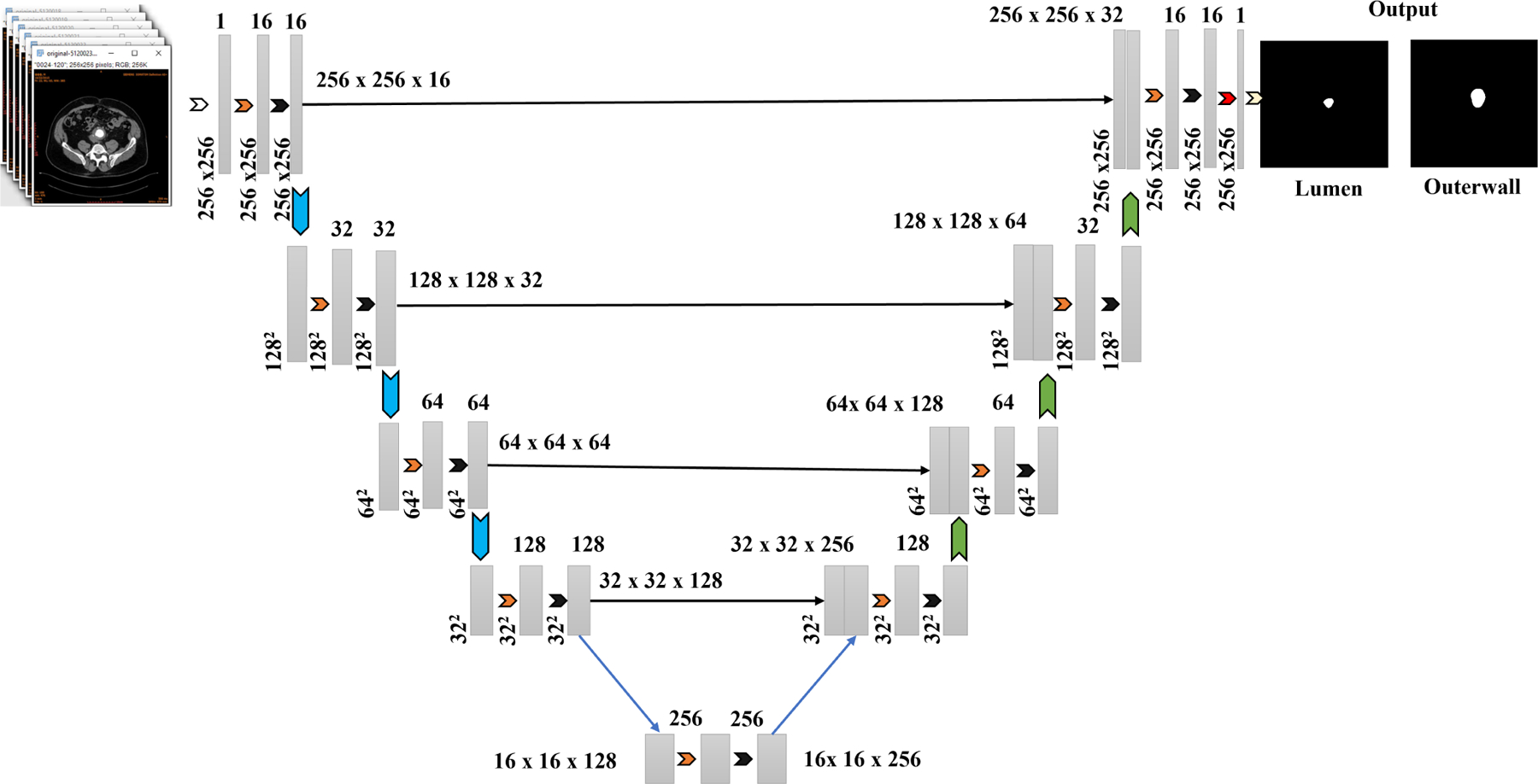
Structural design of the specialized expert U-Net models.

The proposed framework leverages three individually trained U-Net models, designated as Expert 1, Expert 2, and Expert 3. Each of these models is optimized using a unique training setup, with distinct loss functions guiding their learning processes. This diversity enables each expert to specialize in recognizing different structural and textural patterns commonly observed in AAA cases. By including such variation during training, the framework becomes better equipped to handle complex segmentation tasks. Figure 2 shows the structural design of the specialized experts.

At its core, the system uses the U-Net architecture, which is structured as an encoder-decoder design enhanced with skip connections. Within the encoder, the spatial resolution of the input is progressively reduced while hierarchical feature representations are extracted through successive layers involving convolution, ReLU activation, and max pooling. This encoding process is formally described by Equation (1),

(1) $$F_{i+1}=\text{MaxPool}\left(\text{ReLU}\left(\text{Conv}\left(F_{i},W_{i}\right)+b_{i}\right)\right)$$

where $F_{i}$ denotes the feature map at layer i, while $W_{i}$ and $b_{i}$ represent the corresponding weights and biases. The encoder plays a crucial role in capturing the broader context of the input, which is essential in medical imaging applications, such as identifying pathological regions in CT or MRI data.

At the deepest level of the network, the bottleneck layer processes the output from the final encoder stage without further downsampling, as given by Equation (2),

(2) $$B=\text{ReLU}\left(\text{Conv}\left(F_{\text{last}},W_{b}\right)+b_{b}\right)$$

where the final encoder feature map $F_{\text{last}}$ is transformed into a set of abstract representations. These high-level features are particularly valuable for capturing complex anatomical patterns in vascular structures, such as variations in the AAA lumen and wall regions in CTA images.

Next, the decoder reconstructs the spatial resolution by performing upsampling operations and merging the results with the corresponding encoder outputs via skip connections, as shown in Equation (3),

(3) $$U_{i}=\text{Concat}\left(\text{Upsample}\left(D_{i+1}\right),F_{i}\right)$$

where $D_{i+1}$ represents the decoder feature map of the lower level, and $F_{i}$ is the encoder output at the matching resolution. This configuration helps to retain crucial spatial information, such as organ boundaries or vessel contours, which are often lost during downsampling.

The final segmentation map is produced using a 1 × 1 convolution followed by a sigmoid activation function, such as that given by Equation (4),

(4) $$S=\text{Sigmoid}\left({\text{Cpnv}}_{1\times 1}\left(D_{1},W_{f}\right)+b_{f}\right)$$

where the resulting output S is a probability map that highlights the most likely region of interest in a single channel.

Each expert U-Net yields a segmentation mask of dimensions [1, H, W] and the outputs are subsequently stacked to form a tensor of shape [3, H, W]. To encourage specialization, models are trained with varying objectives. Expert 1 utilizes a hybrid of Binary Cross-Entropy (BCE) and Dice loss, offering a balance between pixel-level precision and region-based overlap. Expert 2 adopts Focal Loss, which gives greater attention to hard-to-classify regions, thus addressing class imbalance. Expert 3, although using the same BCE + Dice combination as Expert 1, begins with a different initialization and data partitioning, which prompts it to learn distinct features. Together, this ensemble setup enhances robustness and adaptability, particularly in capturing the varied AAA morphologies, such as differences in the lumen and outer wall boundaries.

#### 3) ERROR MAP COMPUTATION

The predictions of the three expert models are compared with the ground truth mask to compute the pixel-wise error maps, resulting in three separate error maps. These maps highlight regions where each expert fails and are crucial for learning corrective behavior. In Equation (5),

(5) $$E_{i}=\left|P_{i}-Y\right|$$

$E_{i}\left(x,y\right)$ denotes the error at pixel $\left(x,y\right)$ for the i-th expert model, $P_{i}\left(x,y\right)$ is the predicted probability of the i-th model, and $Y\left(x,y\right)$ is the corresponding ground truth label. The resulting error maps $E_{1}$, $E_{2}$, and $E_{3}$ are used as input to the error learner router. Figure II-B3 shows the computation process from the original and predicted masks.

#### 4) ERROR LEARNER ROUTER

This module aims to localize and identify areas of failure in the predictions using a CNN. It takes as input a four-channel tensor formed by concatenating the original input image and the three error maps, which results in shape [4, H, W]. Through a series of convolution layers, it learns to extract spatial patterns of errors and outputs an error feature map of shape [8, 256, 256]. These learned error features are then used to inform the robust weight router by encoding spatial context around misclassified regions such as thin walls, overlapping tissue intensities, and low-contrast zones.

#### 5) ROBUST WEIGHT ROUTER

This component is a 2D convolutional module followed by a softmax activation that generates a probability distribution per pixel in the three expert models. It takes as input the concatenation of the original input and the error features [8, H, W] to produce a combined tensor of shape [9, H, W]. The output of the robust router is a weight map of shape [3, H, W], where each value represents the contribution of an expert to each pixel’s final label. The weights are used for pixel-wise fusion.

#### 6) WEIGHTED PREDICTION FUSION

The framework incorporates a router to dynamically assign regions of the input image to specific U-Net models, as in Equation (6),

(6) $$R=\text{softmax}\left(\text{Conv}\left(X,W_{r}\right)+b_{r}\right)$$

where R represents the routing map, X is the input image, and $W_{r}$ and $b_{r}$ are the weights and biases of the convolutional layer.

Complementing the router, the classifier generates attention maps to highlight complex regions. The attention maps are derived using gradient-based computations given by Equation (7),

(7) $$A_{x}=Z_{i}\cdot \frac{\partial Z_{i}}{\partial X}/{\left\|\frac{\partial Z_{i}}{\partial X}\right\|}_{1}$$

where $Z_{i}$ is the classifier output for class i, and ${\left\|\frac{\partial Z_{i}}{\partial X}\right\|}_{1}$ is the gradient norm.

The segmentation loss is defined by Equation (8),

(8) $${𝓛}_{\text{seg}}=\frac{1}{N}\sum_{i=1}^{N}\left[Y_{i}\cdot \log\left(S_{i}\right)+\left(1-Y_{i}\right)\cdot \log\left(1-S_{i}\right)\right]$$

to evaluate the pixel-wise difference between the predicted segmentation S and the ground truth Y.

To promote diversity among the three U-Net models, the diversity loss is introduced in Equation (9),

(9) $${𝓛}_{\text{div}}=\sum_{i=1}^{m}\sum_{j=i+1}^{m}{\left\|\theta_{i}-\theta_{j}\right\|}_{2}$$

where $\theta_{i}$ and $\theta_{j}$ are the parameter sets of models i and j, and m is the number of models.

In scenarios where the participation of a larger number of experts in the model is required, ${𝓛}_{\text{div}}$ can be approximated by the distance of the parameter between sampled pairs of expert networks, as in Equation (10),

(10) $${\hat{𝓛}}_{\text{div}}=E_{i,j\in \left\{1,\ldots ,m\right\}i\neq j}{\left\|\theta_{i}-\theta_{j}\right\|}_{2}$$

The number of pairs sampled per optimization step can be set to match the computational capability. The total loss combines these objectives (Equation (11)),

(11) $${𝓛}_{\text{total}}={𝓛}_{\text{seg}}-\lambda_{\text{reg}}\cdot {𝓛}_{\text{div}}$$

where $\lambda_{\text{reg}}$ balances segmentation accuracy and diversity. This combination ensures that the framework leverages the strengths of all models while maintaining specialization.

A weighted summation is performed across the three expert predictions using the learned per-pixel weights given by Equation (12),

(12) $$P_{\text{final}}\left(x,y\right)=\sum_{i=1}^{3}w_{i}\left(x,y\right)\cdot P_{i}\left(x,y\right)$$

where $P_{i}\left(x,y\right)$ is the prediction of expert i at pixel $\left(x,y\right)$, and $w_{i}\left(x,y\right)$ is the corresponding learned weight. This fusion results in a final mask prediction of shape [1, H, W], which represents the probability of having an AAA present.

### C. NURBS

In cases of failure (e.g., low-contrast or thin-wall regions), misclassified regions are visualized, and NURBS-based interactive correction can be applied, allowing expert users to adjust contours.

**FIGURE 4.**
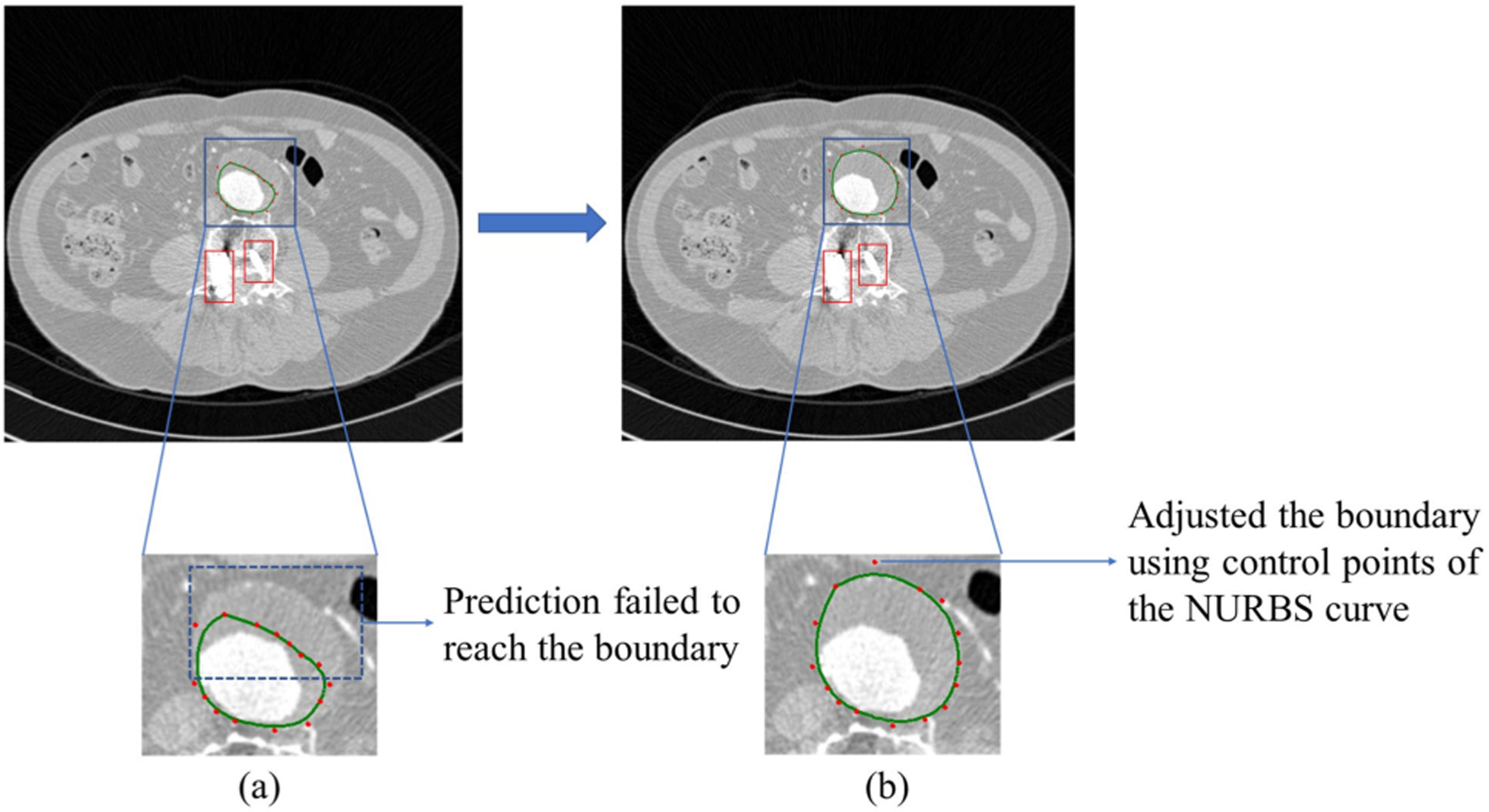
Interactive correction process using NURBS in a frame with significant image artifacts.

When the segmentation model fails to accurately identify boundaries, especially in areas with low contrast or very thin structures, the system shifts to an interactive correction phase. As shown in Figure 4, any regions where the prediction does not align properly are highlighted and presented to the user. This allows an expert to make manual adjustments using a tool called NURBS [17], [46].

The correction is carried out by fitting a smooth curve through the updated control points selected by the user. This is done using the functions splprep and splev from the SciPy library. When the user clicks or drags points onto the canvas, the system records these as coordinate pairs. These points are then passed into a NURBS based function to generate a continuous contour that better matches the desired shape. The curve is calculated using Equation (13),

(13) $$\text{C}\left(u\right)=\sum_{i=0}^{n}N_{i,k}\left(u\right)P_{i}$$

where $P_{i}$ stands for the control points defined by the user, $N_{i,k}\left(u\right)$ are the B-spline basis functions of degree k, and u is a parameter that moves from zero to one along the curve. This setup allows for a smooth and accurate contour that adjusts according to the user’s edits.

After the smoothing step is applied, the updated boundary is drawn on the image to give immediate visual feedback. The corrected result is then saved as a binary mask that can be used for further analysis or evaluation. This approach combines fast automatic segmentation with the flexibility of expert-driven corrections using NURBS, making it useful for medical image tasks where precision is especially important.

### D. EVALUATION METRICS

To rigorously assess the performance of the segmentation model, three widely used metrics were evaluated: IoU, DS, and HD95.

#### 1) INTERSECTION OVER UNION

The IoU, also known as the Jaccard index, measures the overlap between the predicted segmentation mask and the ground truth segmentation mask. IoU is calculated by dividing the intersection area between the predicted and ground truth masks by their union area [27], as shown in Equation (14),

(14) $$\text{IoU}=\frac{\left|A\cap B\right|}{\left|A\cup B\right|}$$

where A is the ground truth segmentation and B is the predicted segmentation. IoU ranges from 0 (no overlap) to 1 (perfect overlap), with higher scores indicating greater accuracy.

#### 2) DICE SIMILARITY COEFFICIENT (DSC)

DSC calculates the similarity between two segmentation masks by measuring their overlap [28], defined mathematically with Equation (15),

(15) $$\text{Dice}=\frac{2\left|A\cap B\right|}{\left|A\right|+\left|B\right|}$$

where A represents the ground truth mask and B the predicted mask. Similarly to IoU, the DSC ranges from 0 to 1, with 1 indicating perfect agreement between the predicted and ground truth segmentations.

#### 3) 95% HAUSDORFF DISTANCE (HD95)

HD95 quantifies the spatial boundary accuracy between two segmentation contours by measuring how far two subsets of a metric space are from each other. HD95 is robust to outliers and represents the 95th percentile of the distances between the boundary points in the predicted segmentation and the ground truth segmentation [29]. It is defined by Equation (16),

(16) $$\text{HD}95=\max\left(h_{95}\left(A,B\right),h_{95}\left(B,A\right)\right)$$

where h95(A, B) denotes the 95th percentile of distances from the boundary points of set A to the closest boundary points of set B, and vice versa. Lower HD95 values indicate more accurate boundary delineation, reflecting better segmentation precision. Collectively, these metrics provide a comprehensive assessment of segmentation quality that addresses both the accuracy of region overlap and boundary precision.

To enhance interpretability and trust in automated segmentation results, the proposed framework explicitly integrates explainability mechanisms within its architecture. These mechanisms comprise specialized expert models trained to identify distinct anatomical complexities, an error learner router that visualizes prediction errors, and a robust weight router that adaptively combines expert predictions through attention maps. Together, these components provide insight into the decision-making process of the black-box model.

## III. RESULTS

### A. QUANTITATIVE ANALYSIS

**FIGURE 5.**
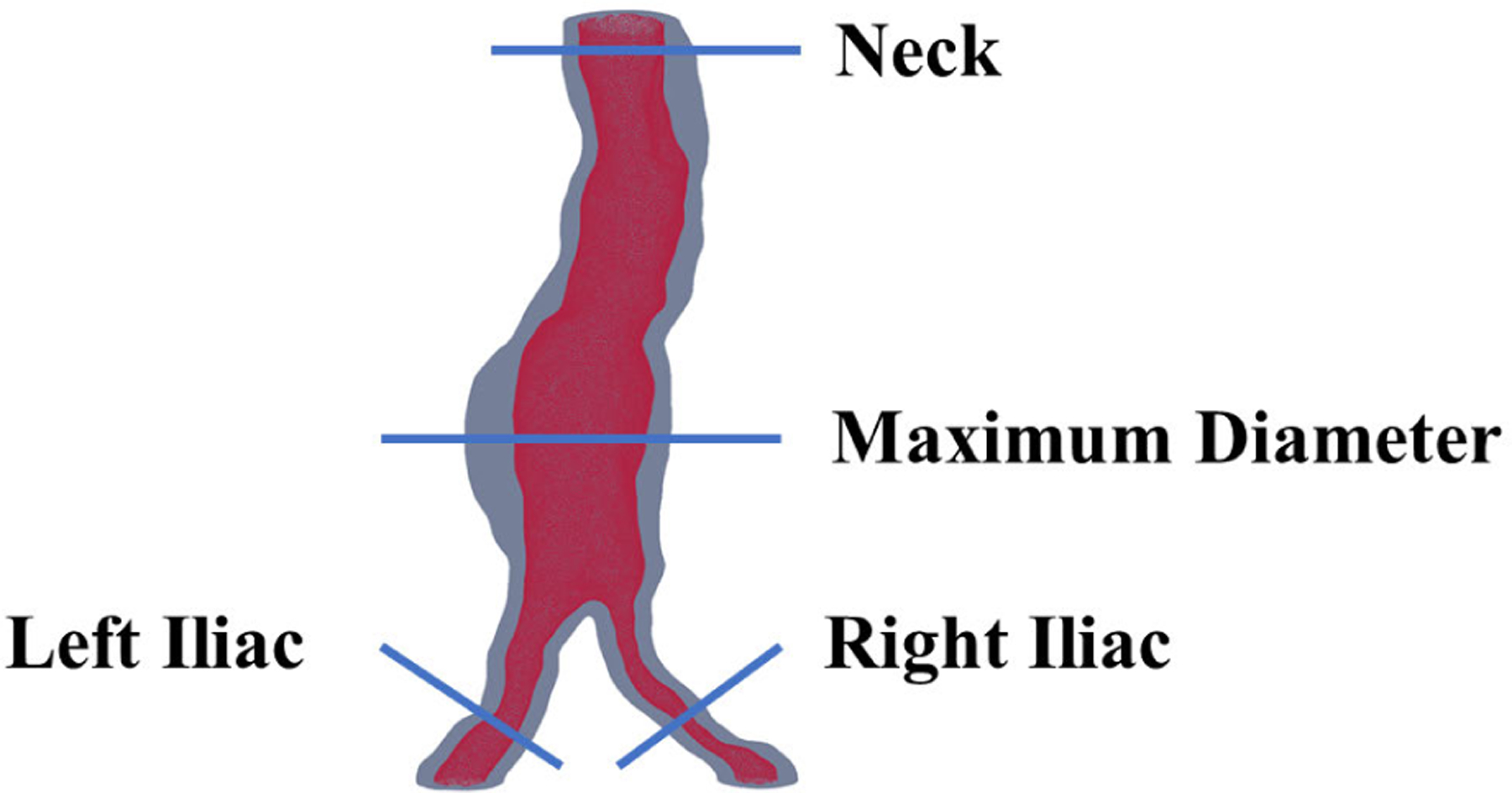
Key cross sections relevant for endovascular graft repair planning, including the neck, maximum diameter, left common iliac, and right common iliac arteries.

To support clinical decision making during AAA repair, the proposed approach focuses on identifying four key anatomical cross sections, as illustrated in Figure 5. These sections are critical for determining the placement and size of the endovascular graft during AAA repair. To evaluate the performance of the model, quantitative analyses were performed with the test data set (1,560 images representative of 22 AAA CTA scans) measuring the lumen area of the neck, the area of the right and left common iliac arteries, and the maximum hydraulic diameter of the outer wall. These metrics ensure that the model provides reliable and accurate data for both diagnostic and interventional planning purposes.

Table 1 shows the performance metrics for the proposed segmentation model, demonstrating its high accuracy (0.9995 and 0.9995) and specificity (0.9648 and 0.9615) for the different lumen and outer wall segmentation tasks, respectively. Sensitivity values of 0.9757 and 0.9754 indicate robust detection capability for AAA regions, while precision scores of 0.9556 and 0.9497 reflect the model’s reliability in correctly segmenting target areas. The DSC (0.9648 and 0.9615) and IoU scores (0.9324 and 0.9264) further highlight the strong performance of the model in delineating accurate boundaries. Furthermore, the high Matthews Correlation Coefficient (MCC) and F-measure values (0.9650 and 0.9618, and 0.9648 and 0.9615, respectively) confirm balanced and consistent results, underscoring the model’s suitability for clinical implementation in medical imaging workflows.

Table 2 shows a comparison between ground truth and predicted geometric measurements for key anatomical regions relevant to AAA assessment. These include the average values of the lumen area of the neck, the maximum diameter measured with the outer wall, and the cross-sectional areas of the two common iliac arteries. The predicted neck lumen area was 3.9717 cm^2^, closely matching the original value of 3.9220 cm^2^. For the outer wall, the predicted maximum diameter was 5.8586 cm, which differed only slightly from the ground truth of 5.8105 cm. For the common iliac artery cross-sections, prediction errors were minimal, typically within ± 0.05 cm^2^. This close alignment highlights the effectiveness of the model in preserving essential anatomical details, supporting its use in accurate preoperative planning, endograft sizing, and patient-specific AAA treatment.

**FIGURE 6.**
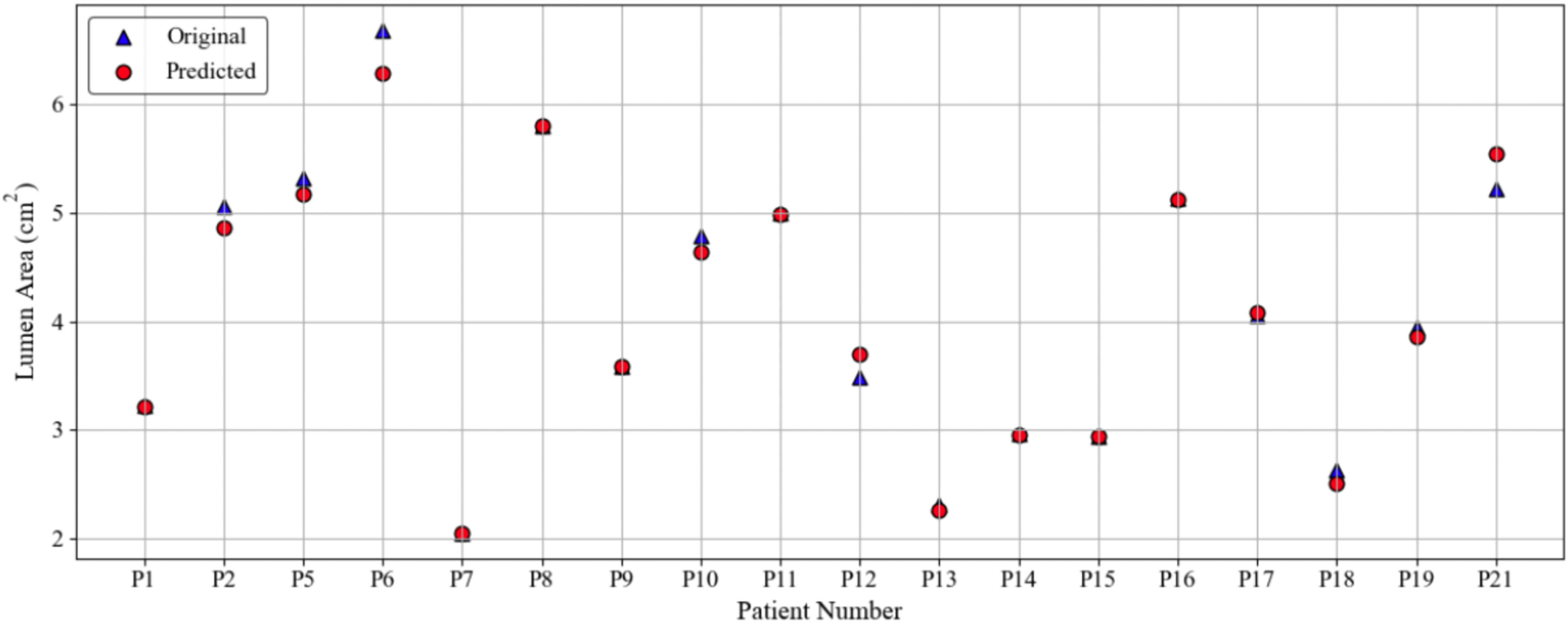
Comparison between original and predicted lumen area measurements at the neck for 21 patients.

Figure 6 shows the comparison between the original and predicted lumen areas at the neck for 21 patients. The predicted measurements closely follow the ground truth values for most AAAs, suggesting that the model accurately replicates the size of the neck lumen for most patients. Some small differences are visible in a few patients, such as P6. The Root Mean Square Error (RMSE) for the 21 discrete measures was 0.1537 cm^2^.

**FIGURE 7.**
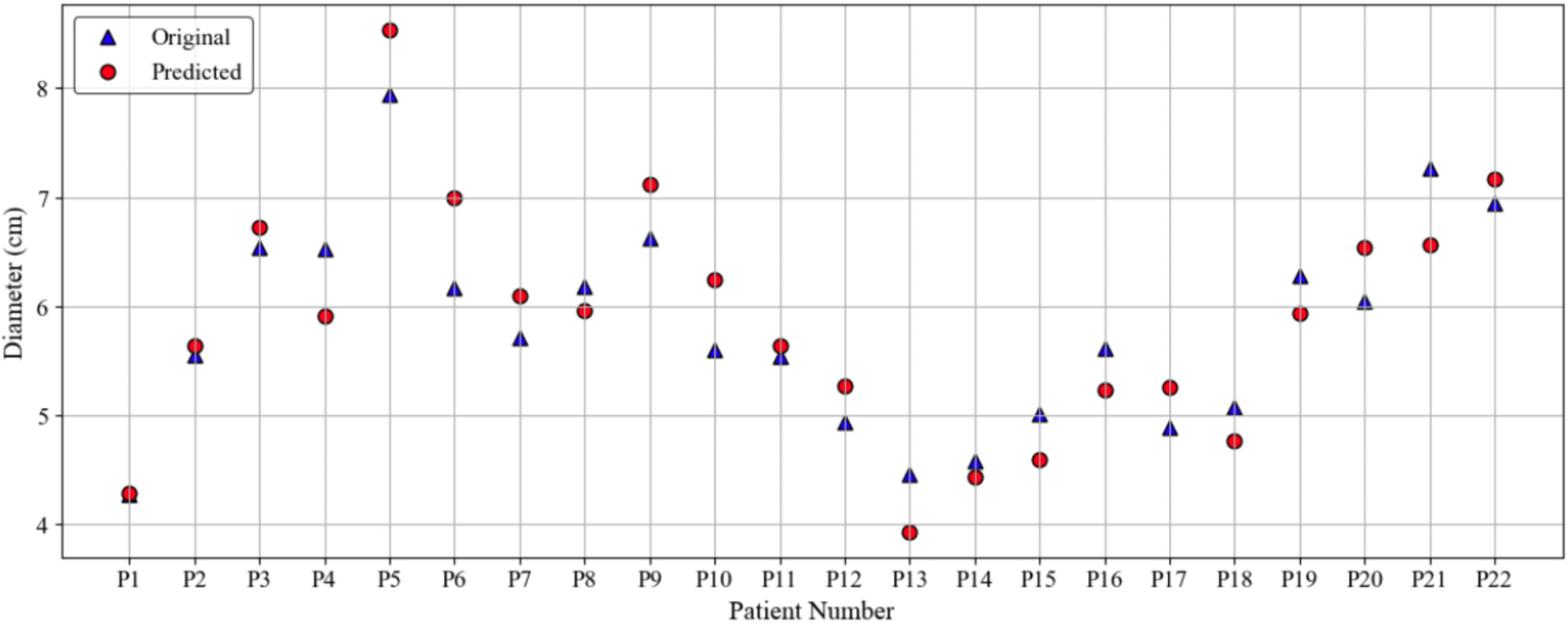
Comparison between original and predicted maximum diameters measured at the outer wall boundary for 22 patients.

Figure 7 shows the comparison between the original and predicted values of the maximum diameter measured using the outer wall boundary in 22 patients. We illustrate the discrete values of maximum diameter for the test data set, showing how the model performs for a wide range of aneurysm sizes. Although most predicted diameters are similar to ground truth measurements, there are some noticeable differences, particularly for patients P6 and P13, where the predicted values appear to be slightly overestimated or underestimated, respectively, compared to the original measurements.

**FIGURE 8.**
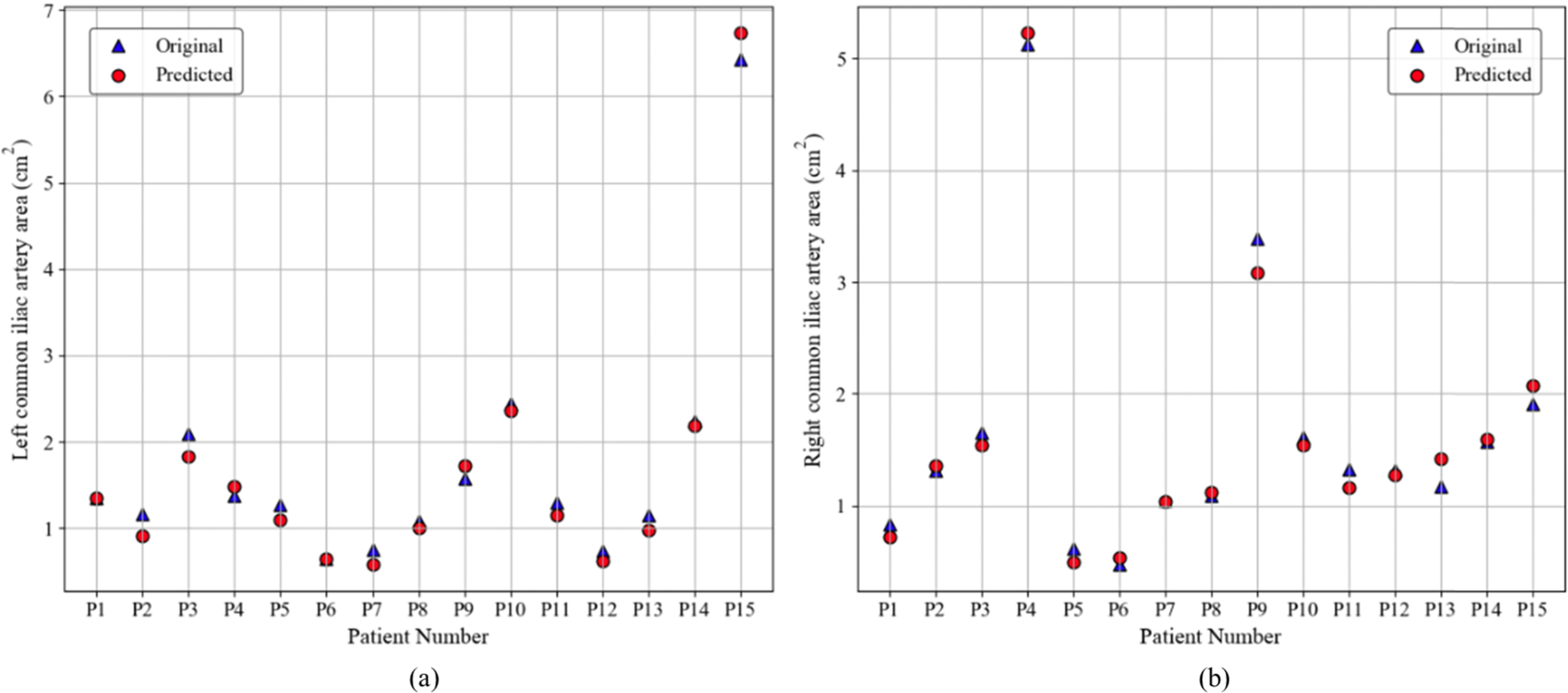
Comparison between the original and predicted lumen areas of the (a) left and (b) right common iliac arteries for 15 patients.

Figure 8 shows a comparison of the original and predicted lumen areas for the left (a) and right (b) common iliac arteries in 15 patients. The two curves are closely aligned, which shows that the predicted measurements follow the same pattern as the actual values. Most predicted areas are similar to the ground truth measurements. Some notable exceptions are patient P15 for the left common iliac and patient P13 for the right common iliac artery. The RMSE for the 15 discrete measures was 0.1599 cm^2^ for the left common iliac and 0.1359 cm^2^ for the right common iliac, reflecting a consistent and accurate representation of the underlying anatomical variations.

### B. EXPLAINABILITY ANALYSIS

The proposed segmentation framework utilizes a Mixture-of-Experts (MoE) strategy composed of specialized expert models. Each expert model is individually trained on distinct subsets of anatomical and textural features observed in AAA CTA images, such as thin vessel walls, variations in contrast intensity, presence of thrombus, and complex geometrical irregularities. By explicitly assigning each expert a distinct set of image characteristics, the system provides inherent interpretability. This approach provides clear insight into the performance of each model in relation to specific anatomical scenarios. For example, one expert might excel at identifying low-contrast boundary areas, while another may specialize in delineating regions with complex textures. To further enhance interpretability, the Error Learner Router systematically analyzes the output of each expert model. Specifically, it generates error maps by comparing each expert’s prediction with the ground truth segmentation masks at a pixel-by-pixel level. These error maps explicitly display spatial regions of segmentation inaccuracies, highlighting precisely where and why each expert model fails or succeeds. Such visual feedback mechanism provides detailed insight into each expert’s limitations, pinpointing problematic anatomical regions such as narrow aneurysm necks, areas of complex curvature, or sections with significant image noise. Consequently, it allows for iterative refinement of training methodologies or preprocessing techniques to address specific weaknesses and improve the overall robustness of the system.

The framework additionally integrates a Robust Weight Router, which dynamically computes per-pixel attention weights based on the learned error features from the Error Learner Router. These attention weights indicate the degree of trustworthiness assigned to each expert model’s prediction on a pixel-by-pixel basis. Using convolutional neural network layers followed by a softmax activation, the router derives an interpretable attention map that explicitly quantifies each expert’s contribution to the final segmentation. The attention map transparently visualizes areas where certain experts dominate due to their specialized expertise. This intuitive visualization significantly improves interpretability, validation, and confidence in the automated decisions made by the model. The final interpretative component, Weighted Prediction Fusion, synthesizes the predictions of individual experts into a single coherent segmentation mask. This fusion is accomplished through a pixel-wise weighted summation, using the attention weights computed by the Robust Weight Router. By providing explicit weighting at each pixel, the methodology allows us to trace and understand the exact reasoning behind the final segmentation output of the model. This step effectively demystifies the traditionally opaque nature of deep learning-based segmentation; it offers a detailed insight into which expert influenced each region of the segmentation result. The process delivers complete transparency, allowing a precise assessment of segmentation quality and quick identification of areas requiring further adjustments or attention.

Collectively, these integrated explainability mechanisms significantly increase the usability and reliability of the segmentation framework. By providing comprehensive visual analytics tools including expert specialization maps, explicit error visualizations, detailed attention-weight mappings, and interpretable fusion strategies, the framework offers thorough and intuitive methods for critically evaluating and confidently utilizing automated AAA segmentation outcomes.

## IV. DISCUSSION

**FIGURE 9.**
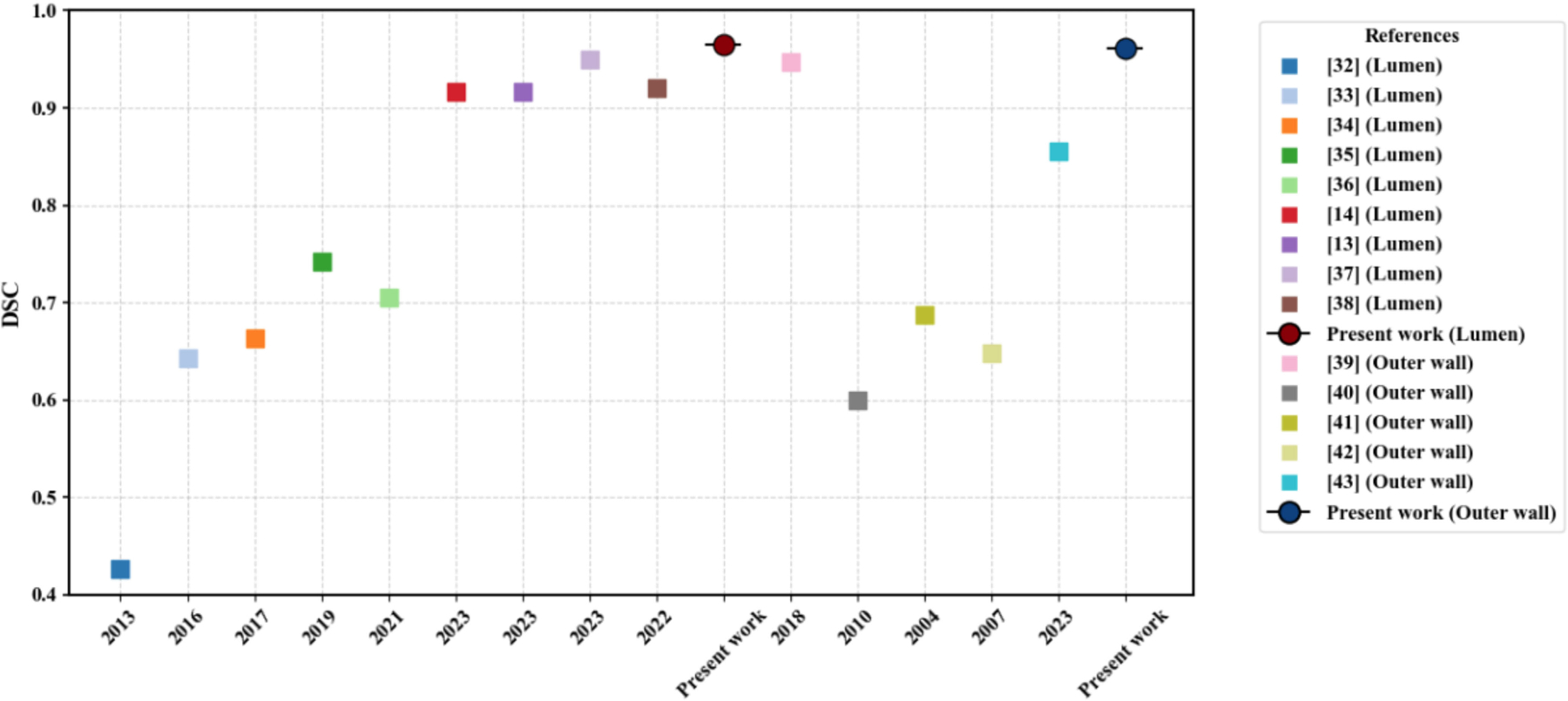
DSC scores reported by various segmentation methods. Each marker corresponds to a different method as specified in the legend.

The proposed explainable AI-based segmentation framework for AAA detection demonstrates superior performance by addressing critical limitations of existing methodologies. Traditional approaches, such as fully automated pipelines for AAA segmentation, have achieved significant milestones in efficiency but often face challenges in precision, particularly in delineating complex anatomical structures, as noted by Lareyre et al. [23]. Although such pipelines provide foundational insight, their limited focus on advanced boundary modeling affects the accuracy of the predictions made from their implementation in clinical scenarios. To this end, Figure 9 shows the DSC reported by various segmentation methods published between 2013 and 2024, categorized by lumen and outer wall segmentation. Each colored square corresponds to a specific reference, as specified in the legend. Although several existing methods show DSC values ranging from approximately 0.6 to just above 0.9, the results vary noticeably for all the techniques. The proposed framework achieved the highest DSC for lumen and outer wall segmentation (0.9648 and 0.9615), emphasizing the method’s higher and more consistent segmentation accuracy compared to existing approaches.

A further comparison of the proposed framework with previous methods is summarized in Table 3. We describe a comparative analysis of HD95 values for various 2D segmentation models applied to distinct medical imaging modalities, given the lack of HD95 data available in the literature for AAA CTA segmentation. For esophagus CT segmentation, the Two-Step DL Network reported by Zhang et al. [43] achieved an HD95 of 4.32 mm, indicating moderate accuracy in edge delineation. For ovarian tumor CT segmentation, U-Net and its extensions (UNet++, TransUNet, and Swin-UNet) produced HD95 scores ranging from 22.35 mm to 34.43 mm, reflecting difficulties in managing irregular tumor boundaries [44]. For prostate MRI segmentation, the standard U-Net achieved an HD95 of 4.04 mm, while alternatives such as DRUNet, SegResNet, and Swin-UNet achieved marginal gains with HD95 ranaging from 3.85 mm to 3.21 mm, likely due to improved spatial feature modeling and attention mechanisms [45]. The proposed framework produced lower HD95 values of 1.3490 mm for the lumen and 1.3670 mm for the outer wall, although based on AAA CTA images. Despite the difference in data sets, this comparison demonstrates the high precision of the present method in anatomical boundary localization, exceeding the existing models’ boundary-level accuracy.

The framework incorporates advanced architectural features, such as attention-based classifiers and NURBS-based boundary corrections, to achieve precise segmentation of the AAA lumen and outer wall. These enhancements are crucial for subsequent three-dimensional shape reconstruction and stress analysis, aligning with methodologies that emphasize shape accuracy and wall stress evaluations, as demonstrated by Shim et al. [26] and Mesri et al. [30]. In addition to strong numerical results, the proposed framework has clear advantages for eventual clinical translation. It can accurately outline complex anatomical structures, even when image quality changes due to differences in contrast, scanner types, or the presence of artifacts. This shows that the model performs well under a variety of imaging conditions. It also includes explainable features, such as attention maps, which make the results more transparent and easier for clinicians to interpret. These feature descriptions would allow medical professionals to verify and understand how the model arrived at its predictions. This added clarity supports better decision making and risk assessment of AAAs in a clinical setting. Delayed adverse reactions and image artifacts in contrast-enhanced CT scans, as highlighted by Loh et al. [24], complicate segmentation tasks and emphasize the need for robust frameworks that can manage variability in imaging conditions. Singh et al. [25] postulated that the integration of biomechanical parameters, such as peak wall stress (PWS) and rupture risk indices, can significantly improve the predictive accuracy of such frameworks and, intuitively, clinical outcomes. Therefore, future work in this field should incorporate the use of biomechanical quantification of AAA rupture risk based on computer models created with the proposed explainable AI-based segmentation framework. Such work would build on recent advances in AI for AAA risk assessment, as highlighted by Chung et al. [31]. While the proposed explainable segmentation framework demonstrates robust performance, several limitations and edge cases require consideration. The model accuracy may be reduced in low-contrast CTA images or cases with significant artifacts, such as those caused by patient motion or suboptimal contrast administration, which were underrepresented in the current dataset. Additionally, rare AAA morphologies or thin vessel walls not captured well in the training set could pose challenges to segmentation precision. The reliance of the framework on the diversity and quality of training data suggests that its generalizability may be limited for diverse populations. Future enhancements, such as data augmentation or multicenter validation, could mitigate these issues.

## V. CONCLUSION

This study introduces a DL-based segmentation framework designed to identify and delineate the AAA lumen and outer wall boundaries in CTA images. By combining specialized U-Net models, a dynamic routing mechanism, and a robust model fusion strategy, the system effectively segments the lumen and outer wall with high accuracy. It achieves strong quantitative performance, with DSC values of 0.964 and 0.962, IoU scores of 0.921 and 0.919, and HD95 values of 1.3490 mm and 1.3670 mm for the lumen and the outer wall, respectively. The integration of NURBS-based corrections improves its ability to manage complex boundary variations in difficult cases. Although the fully automated system processes each image frame in approximately 17 ± 0.02 milliseconds, the optional manual adjustment using NURBS in challenging cases requires an average of 3 to 20 seconds per frame. As the accuracy of the framework depends on the diversity and quality of the training data, we recommend expanding the data set to include broader demographics and clinical variability. Future research should focus on expanding the dataset to enhance generalizability across diverse populations and imaging conditions. In addition, integrating biomechanical modeling, such as wall stress estimation, could improve the assessment of rupture risk based on computer models generated from the use of this framework. Further optimization of the NURBS refinement process is expected to reduce correction times and enhance efficiency, building on the framework’s current capabilities. Nevertheless, this framework provides a reliable and efficient foundation to develop intelligent tools that can improve AAA diagnosis and treatment planning in clinical practice.

## Figures and Tables

**FIGURE 3.**
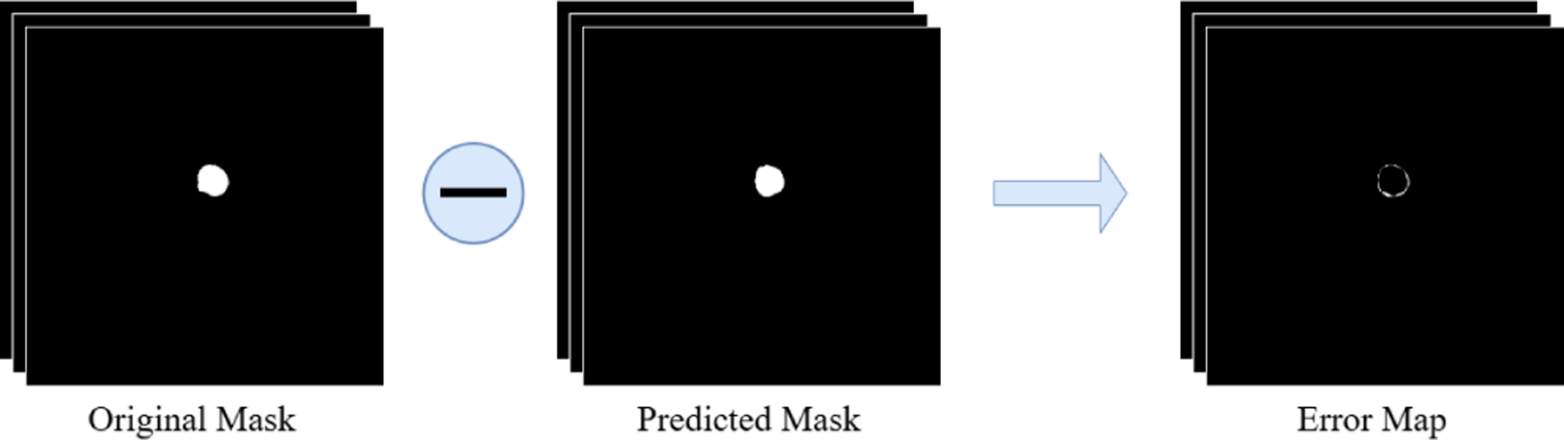
Error map computation process from original and predicted masks.

**TABLE 1.** Performance metrics for fully automatic segmentation model.

Metric	Value (Lumen)	Value (Outer Wall)
Accuracy	0.9995	0.9995
Sensitivity	0.9757	0.9754
Precision	0.9556	0.9497
F-measure	0.9648	0.9615
MCC	0.9650	0.9618
DSC	0.9648	0.9615
Specificity	0.9996	0.9996
IoU	0.9324	0.9264

**TABLE 2.** Geometric measurements: original vs. predicted values.

Metrics	Values
Neck - Lumen Area (cm^2^) - Original	3.9220
Neck - Lumen Area (cm^2^) - Predicted	3.9717
Outer Wall - Maximum Diameter (cm) - Original	5.8105
Outer Wall - Maximum Diameter (cm) - Predicted	5.8586
Right Common Iliac - Lumen Area (cm^2^) - Original	1.7031
Right Common Iliac - Lumen Area (cm^2^) - Predicted	1.6627
Left Common Iliac - Lumen Area (cm^2^) - Original	1.6302
Left Common Iliac - Lumen Area (cm^2^) - Predicted	1.6739

**TABLE 3.** Performance comparison of 2D segmentation methods using HD95.

Model / Method	Dataset / Task	HD95 (mm)	Reference [No.]
Two-Step DL Network	Esophagus Segmentation (CT)	4.32 ± 1.02	[43]
U-Net	Ovarian Tumor Segmentation (CT)	27.63	[44]
UNet++	Ovarian Tumor Segmentation (CT)	34.43	
TransUNet	Ovarian Tumor Segmentation (CT)	22.35	
Swin-UNet	Ovarian Tumor Segmentation (CT)	30.80	
2D U-Net	Prostate Segmentation (MRI)	4.04	[45]
2D DRUNet	Prostate Segmentation (MRI)	3.85	
2D SegResNet	Prostate Segmentation (MRI)	3.36	
2D Swin-UNet	Prostate Segmentation (MRI)	3.28	
2D TransUNet	Prostate Segmentation (MRI)	3.21	
**Proposed Method (Lumen)**	AAA Segmentation (CTA)	**1.3490**	-
**Proposed Method (Outer Wall)**	AAA Segmentation (CTA)	**1.3670**	-
